# Sciatic Neuralgia

**Published:** 1878-03

**Authors:** 


Sciatic Neuralgia.—I was called to see Fred W., aged 31, on January 1, 1878. Found him suffering intensely from neuralgia of the great sciatic nerve. Learned from him that he had suffered more or less from this disease for about a year. Had taken quinia, strychnia, and morphia, internally, the latter to such extent as to render it difficult to do without it. He had used stimulating and anodyne applications locally as well as a series of blisters over the course of the nerve. I commenced the treatment of this case by using the continued current over the course of the nerve, from its origin to its termination, on the outside of the foot. After second application of the battery improvement began, but as the pain persisted to an uncomfortable degree in the region of the popliteal space, after the fourth application of the battery I injected one forty-eighth of a grain of sulphate of atropia, hypodermically, over the seat of the pain. In a short time the pain was gone, and has not returned since. The hypodermic injections of sulphate atropia, in one forty-eighth grain doses, were repeated two or three times, as was the galvanism also. I consider this case of sufficient interest to merit a report: 1. Because of the comparative long duration of the disease without getting any relief to speak of, the patient having been under the care of a physician all the while, and for the four weeks previous to my first visit, confined to the house, and to the bed for most part. 2. The prompt response to the remedies made use of. Which of the remedies, the electricity or the atropia, had the most to do in bringing about the result above stated, we leave it to the readers of the *Reporter* to judge; however, I believe that the electricity would have effected a cure after a little time, but that the atropia materially hastened the desired result.—*Medical and Surgical Reporter.*

